# The Antioxidative Role of Natural Compounds from a Green Coconut Mesocarp Undeniably Contributes to Control Diabetic Complications as Evidenced by the Associated Genes and Biochemical Indexes

**DOI:** 10.1155/2021/9711176

**Published:** 2021-07-27

**Authors:** Rickta Rani Das, Md. Atiar Rahman, Salahuddin Qader Al-Araby, Md. Shahidul Islam, Md. Mamunur Rashid, Nouf Abubakr Babteen, Afnan M. Alnajeebi, Hend Faisal H. Alharbi, Philippe Jeandet, Md. Khalid Juhani Rafi, Tanvir Ahmed Siddique, Md. Nazim Uddin, Zainul Amiruddin Zakaria

**Affiliations:** ^1^Department of Biochemistry and Molecular Biology, University of Chittagong, Chittagong 4331, Bangladesh; ^2^Department of Biochemistry, Collage of Science, University of Jeddah, Jeddah 80203, Saudi Arabia; ^3^Department of Food Science and Human Human Nutrition, Collage of Agriculture and Veterinary Medicine, Qassim University, Buraydah, Saudi Arabia; ^4^Department of Biology and Biochemistry, Faculty of Sciences, University of Reims, PO Box 1039, Reims, France; ^5^Institute of Food Science and Technology, Bangladesh Council of Scientific and Industrial Research, Dhaka 1205, Bangladesh; ^6^Department of Biomedical Science, Faculty of Medicine and Health Sciences, Universiti Putra Malaysia (UPM), Serdang, 43400 Selangor, Malaysia

## Abstract

The purpose of this study was to look into the effects of green coconut mesocarp juice extract (CMJE) on diabetes-related problems in streptozotocin- (STZ-) induced type 2 diabetes, as well as the antioxidative functions of its natural compounds in regulating the associated genes and biochemical markers. CMJE's antioxidative properties were evaluated by the standard antioxidant assays of 1,1-diphenyl-2-picrylhydrazyl (DPPH), superoxide radical, nitric oxide, and ferrous ions along with the total phenolic and flavonoids content. The *α*-amylase inhibitory effect was measured by an established method. The antidiabetic effect of CMJE was assayed by fructose-fed STZ-induced diabetic models in albino rats. The obtained results were verified by bioinformatics-based network pharmacological tools: STITCH, STRING, GSEA, and Cytoscape plugin cytoHubba bioinformatics tools. The results showed that GC-MS-characterized compounds from CMJE displayed a very promising antioxidative potential. In an animal model study, CMJE significantly (*P* < 0.05) decreased blood glucose, serum alanine aminotransferase (ALT), aspartate aminotransferase (AST), creatinine, uric acid, and lipid levels and increased glucose tolerance as well as glucose homeostasis (HOMA-IR and HOMA-b scores). The animal's body weights and relative organ weights were found to be partially restored. Tissue architectures of the pancreas and the kidney were remarkably improved by low doses of CMJE. Compound-protein interactions showed that thymine, catechol, and 5-hydroxymethylfurfural of CMJE interacted with 84 target proteins. Of the top 15 proteins found by Cytoscape 3.6.1, 8, *CAT* and *OGG1* (downregulated) and *CASP3*, *COMT*, *CYP1B1*, *DPYD*, *NQO1*, and *PTGS1* (upregulated), were dysregulated in diabetes-related kidney disease. The data demonstrate the highly prospective use of CMJE in the regulation of tubulointerstitial tissues of patients with diabetic nephropathy.

## 1. Introduction

Diabetes mellitus (DM), a metabolic disorder characterized by hyperglycemia induced by insulin secretion deficiency and/or resistance to its action, affects more than millions of people across the world [1]. DM impairs several nonenzymatic and enzymatic antioxidative defense mechanisms that lead to cause oxidative stress as well as tissue damage in DM-associated comorbidities such as cataracts, neuropathy, nephropathy, and retinopathy [2]. Until now, no single effective treatment for DM has been developed in medicine, and the current therapeutic supports such as biguanides, sulfonylureas, meglitinides, thiazolidinediones, dipeptidyl peptidase IV inhibitors, and *α*-glucosidase inhibitors and their analogs have many side effects, such as weight gain, hypoglycemia, gastrointestinal disorders, liver and kidney damage, and hypersensitivity reactions [2, 3].

The above-mentioned side effects suggest that further development of new, safer, and more powerful oral antihyperglycemic agents, particularly in long-term therapy, is needed. In this context, medicinal plants have emerged as promising adjuvants to treat chronic, oxidative stress-mediated disorders [3]. Several medicinal plants recommended for the treatment of DM have been shown to protect *β*-cells, increase insulin secretion and glucose absorption by the adipose tissue, and decrease glucose absorption in the intestines [2, 4]. Some experiments have shown in recent years that most plants produce carotenoids, flavonoids, terpenoids, alkaloids, glycosides, and anthocyanin that exert a significant impact on diabetes and other chronic diseases as well as minimize oxidative stress [5]. Treatments which involve the use of medicinal plants provoking antioxidative actions are therefore highly recommended [6, 7].

Different parts of coconut have long been used as one of the most popular edible foods in almost every part of the world. Nutritional and medicinal values of coconuts have been investigated, and especially, their antibacterial, antihypertensive, oral microflora inhibitory, antiviral, antifungal, antidermatophytic, antiparasitic, hypoglycemic, immunostimulant, and hepatoprotective properties are reported by many scientists [8–10]. Microminerals and nutrients of coconut water are essentially important for human health while the endocarp part is cited to contain high contents of phenolic and flavonoids. Coconut milk has also been shown effective in the management of diabetes [10]. Interestingly, the mesocarp part of coconut has not yet been studied, and we tried here to investigate the antioxidative effect of coconut mesocarp juice which eventually and undeniably contributes to the management of diabetes and renal diabetic complications using a fructose-fed streptozotocin-induced diabetic rat model. The observed effect has been verified and networked with the genes linked in reducing oxidative stress in the biological system and through bioinformatics-based network pharmacological approach in a computational model.

## 2. Materials and Methods

### 2.1. Collection of Coconut Mesocarp

Green coconut mesocarps were collected from the local green coconut seller around the University of Chittagong. The mesocarp juice was extracted using a mechanical sugarcane juicer machine (detailed in the extraction section) with the aid of a local sugarcane juice seller. The mesocarp part of coconut has been keenly identified with the help of a plant scientist Dr. Sheikh Bokhtear Uddin, Professor, Department of Botany, University of Chittagong. A sample specimen of collected mesocarp has been preserved in the institutional herbarium with an identification number (MPSS2017/02).

### 2.2. Chemicals and Reagents

All the chemicals and reagents used in this study were of analytical grade unless specified. ABTS (2,2′-azino-bis(3-ethylbenzthiazoline-6-sulfonic acid)), dinitrosalicylic acid, Folin-Ciocalteu reagent, dimethyl sulfoxide (DMSO), 1,1-diphenyl-2-picrylhydrazyl (DPPH), nitro blue tetrazolium (NBT), potassium ferric cyanide, sodium hydroxide, trichloroacetic acid (TCA), nitroprusside, N-(1-naphthyl) ethylene diamine dihydrochloride, *O*-phenanthroline, and *α*-amylase were procured from Sigma-Aldrich Co., St. Louis, USA. Butanol, n-hexane, methanol (absolute), ethanol (99.99%), and acetone were purchased from Sigma-Aldrich.

### 2.3. Preparation of Coconut Mesocarp Juice Extract (CMJE)

Coconut mesocarp juice extract was prepared as previously described by Rahman et al. [11]. Briefly, the green coconut's mesocarp, which is also known as coir, is situated just beneath the exocarp or outer skin of the fruit. The exocarp part was plucked, and the mesocarp was removed. The liquid sap of the mesocarp (coir) was then mechanically collected. The collected sap was filtered by using filter paper (Whatman filter paper #1) and a funnel. The filtered sap was then evaporated by an electromantle at 45-50°C for several days. The sample collected from the electromantle was further evaporated through a rotary evaporator (RE 200, Bibby Sterilin Ltd., UK) at 55-60°C, and the final extract was collected and stored in the refrigerator.

### 2.4. Screening for Phytochemical Content of CMJE

#### 2.4.1. Total Flavonoid Content (TFC) and Total Phenolic Content (TPC) Determinations

The total flavonoid content (TFC) of CMJE was determined according to the method established by Kumaran and Karunakaran [12]. The total phenolic content (TPC) of the CMJE was measured according to a method described by Singleton and Rossi [13].

#### 2.4.2. Gas Chromatography-Mass Spectroscopy (GC-MS) Analysis of CMJE

The crude CMJE was analyzed by GC-MS using electron impact ionization (EI) with a gas chromatograph (GC-17A, Shimadzu Corporation, Kyoto, Japan) coupled to a mass spectrometer (GC-MS TQ 8040, Shimadzu Corporation, Kyoto, Japan). A fused silica capillary column (Rxi-5ms; 0.25 m film thickness) is coated with DB-1 (J&W). The inlet temperature of the capillary was set at 260°C, and the oven temperature was set at 70°C (0 min), 10°C and 150°C (5 min), 12°C and 200°C (15 min), and 12°C and 220°C (5 min). The column flow rate was 0.6 mL/min of helium gas at a constant pressure of 90 kPa. The auxiliary (GC to MS interface) temperature was set at 280°C. The MS was set in scan mode with a scanning range of 40-350 amu. The mass range was set in the range of 50-550 m/z. The prepared sample was then run for GC-MS analysis. The total GC-MS running time was 35 minutes. All peak areas were compared with the database in the GC-MS library version NIST 08-S.

#### 2.4.3. Estimation of Beta-Carotene and Lycopene Contents of CMJE

Beta-carotene and lycopene contents of CMJE were estimated using a slightly modified method of that described by Kumari et al. [14]. Briefly, 100 mg of the extract was mixed with 10 mL of the acetone-hexane mixture (4 : 6) for 1 minute and filtered. The absorbance was measured at three different wavelengths (453, 505, and 663 nm).

The beta-carotene and lycopene contents were calculated as follows:

Beta-carotene: (mg/100 mL) = (0.216 × *A*_663_) − (0.304 × *A*_505_) + (0.452 × *A*_453_)

Lycopene: (mg/100mL) = −(0.0458 × *A*_663_) + (0.372 × *A*_505_) − (0.0806 × *A*_453_).

#### 2.4.4. Determination of the DPPH Free Radical Scavenging Activity of CMJE

The DPPH free radical scavenging effect was measured according to the method of Shen et al. [15] supplemented with the established protocol described by Brand-Williams et al. [16]. Ascorbic acid was used as a reference antioxidant agent in this experiment. The required amount (0.96 mg) of ascorbic acid and CMJE sample was individually dissolved in 12 mL methanol to prepare stock solution. The stock solution of both CMJE and ascorbic acid was diluted to the concentrations of 40, 20, 10, and 5 *μ*g/mL. Two milliliters of both CMJE solution and ascorbic acid solution of different concentrations was taken as triplicate into test tubes where 2 mL of the freshly prepared DPPH solution was added. The reaction mixture was incubated in the dark for 30 min at room temperature, and the absorbance of the reaction mixture was measured at 517 nm by using a visible spectrophotometer. Control was prepared in similar manner excluding sample.

The percentage of inhibition was calculated by the following equation:

(1) $$\begin{array}{c} \%\text{Inhibition}=\left\{\frac{A_{0}-A_{1}}{A_{0}}\right\}\times 100 \end{array}$$

where *A*_0_ is the absorbance of control (freshly prepared DPPH solution) and *A*_1_ is the absorbance of extract/Std. Then, the percentage of scavenging activity or inhibition was plotted against the concentration, and IC_50_ was calculated by the linear regression analysis from the graph.

#### 2.4.5. Determination of ABTS Radical Scavenging Activity of CMJE

The ABTS free radical scavenging activity of CMJE was measured by using the ABTS (2,2′-azino-bis(3-ethylbenzthiazoline-6-sulfonic acid)) radical cation decolorization assay [17]. ABTS•+ was generated by reacting 7 mM ABTS aqueous solution with 2.45 mM potassium persulfate in the dark for 12-16 h at room temperature. At the beginning of the assay, this solution was diluted in ethanol (about 1 : 49, *v*/*v*) and equilibrated at 30 ± 2°C to give an absorbance of 0.7 ± 0.02 at 734 nm. The stock solution of the CMJE extract was diluted to yield a concentration range of 50-8000 *μ*g/mL. The final concentration (0-15 *μ*M) was obtained by the addition of 1 mL of the diluted ABTS•+ solution to 62 *μ*L of CMJE sample in ethanol. After 40 minutes of mixing, absorbance was estimated at 25°C. Trolox and ethanol were used as a positive control and blank, respectively. At each dilution of the standard and sample, triplicate determinations were made, and absorption was measured at 734 nm in the UV-Vis spectrophotometer (UV-1200S UV-VIS 1200, Shimadzu Corporation, Japan). The ABTS•+ scavenging capability of the extract was compared with that of Trolox. The percentage inhibition calculated as follows:

(2) $$\begin{array}{c} \text{ABTS radical scavenging activity}\left(\%\right)=\left[\frac{\left(\text{Absorbance of control}–\text{Absorbance of the sample}\right)}{\text{Absorbance of control}}\right]\times 100 \end{array}$$

#### 2.4.6. Determination of Superoxide Scavenging Activity of CMJE

The superoxide radical scavenging power of CMJE was assessed by an updated protocol of Rana et al. [18]. Using alkaline dimethyl sulfoxide (DMSO), the superoxide radical was formed by dissolving 250 *μ*L of 1 M NaOH in double-distilled water to 49.750 *μ*L of DMSO. With a NaOH concentration of 5 mM and a volume of 50 mL, air bubbled through the mixture for 1 h and 30 minutes. The solution of NBT (nitro blue tetrazolium) was prepared by dissolving 12 mg of NBT in 12 mL of double-distilled water (pH 7.4), with a final concentration of NBT of 1 mg/mL. The sample was diluted, and each test tube received a volume of 43 *μ*L of each sample, where the sample concentration ranged from 25 to 800 *μ*g/mL. Furthermore, 143 *μ*L of alkaline DMSO and 14 *μ*L of NBT (1 mg/mL) were added to each test tube, incubated for 20 minutes and read at 560 nm for absorbance. DMSO and ascorbic acid were used as negative and positive controls, respectively. Triplicates were confirmed for each experiment. The percentage inhibition calculated as follows:

(3) $$\begin{array}{c} \text{Superoxide radical scavenging activity}\left(\%\right)=\left[\frac{\text{sample Absorbance}-\text{control Absorbance}}{\text{sample Absorbance}}\right]\times 100 \end{array}$$

#### 2.4.7. Estimation of Nitric Oxide Scavenging Activity of CMJE

The nitric oxide scavenging effect was estimated based on the principle of the analysis of nitrite ions which are generated from sodium nitroprusside through nitric oxide in an aqueous solution at a physiological pH [19]. Nitric oxide scavengers compete with oxygen, resulting in decreased nitrite ion production. For the experiment, 1.5 mL of sodium nitroprusside (10 mM) in phosphate-buffered saline (pH 7.4) was combined with various 100 *μ*L volumes of water-dissolved CMJE extracts and incubated for 150 min at room temperature. Without CMJE, the same reaction mixture, but an equal amount of water, served as control. 1.5 mL of the Griess reagent, 1% sulfanilamide, 2 percent H_3_PO_4_, and 0.1 percent N-(1-naphthyl) ethylene diamine dihydrochloride, was added after the incubation time. At 546 nm against the blank, the absorbance of the chromophore formed was read. Ascorbic acid was used as a positive control. The nitric oxide radical scavenging power was calculated by the following formula:

(4) $$\begin{array}{c} \text{Scavenging activity of nitric oxide radical }\left(\%\right)=\left[\frac{\text{Control OD}-\text{Sample OD}}{\text{Control OD}}\right]\times 100 \end{array}$$

#### 2.4.8. Determination of the Iron-Chelating Activity of CMJE

The iron-chelating activity of CMJE was measured using the method of Benzie and Strain [20]. The theory is based on the formation of, and destruction of, the *O*-phenanthroline-Fe^2+^ complex in the presence of chelating agents. A reaction mixture containing 1 mL of 0.05% *O*-phenanthroline in methanol, 2 mL of fresh ferrous chloride (200 *μ*M), and 2 mL of different CMJE concentrations was incubated at room temperature for 10 min, and absorbance was measured at 510 nm. Experiments were performed in triplicate, and the operation was correlated with the usual positive control, ascorbic acid.

(5) $$\begin{array}{c} \text{Inhibition of Iron radical }\left(\%\right)=\left[A_{0}-A_{1}\right]/A_{0}\times 100 \end{array}$$

where *A*_0_ is the test absorbance and *A*_1_ is the control absorbance.

### 2.5. Determination of the *α*-Amylase Inhibition Capacity of CMJE

The *α*-amylase inhibitory action of CMJE was determined by a modified procedure of McCue et al. [21, 22]. Briefly, 4 mg CMJE was dissolved in 5 mL water to prepare a stock solution of 800 *μ*g/mL, which was diluted to 50, 100, 200, and 400 *μ*g/mL. Four milligrams of acarbose (standard) was dissolved in 5 mL water to prepare similar concentrations of standard solutions as was done for CMJE sample. 250 *μ*L of CMJE was mixed in a tube with 250 *μ*L of 0.02 M sodium phosphate buffer (pH 6.9) containing the *α*-amylase solution (0.5 mg/mL). This solution was preincubated at 25°C for 10 min, after which 250 *μ*L of 1% starch solution in 0.02 M sodium phosphate buffer (pH 6.9) was added at timed intervals and then further incubated at 25°C for 10 min. Termination of the reaction was ensured by adding 500 *μ*L of the dinitrosalicylic acid (DNS) reagent. The assay mixtures were then incubated for 5 min in boiling water and cooled to room temperature. The reaction mixture was diluted with 5 mL distilled water, and the absorbance was measured at 540 nm using a spectrophotometer (UV-1280, UV-Vis spectrophotometer, Shimadzu Corporation, Japan). A control was prepared using the same procedure replacing the extract with distilled water. The *α*-amylase inhibitory activity was calculated as percentage inhibition:

(6) $$\begin{array}{c} \%\text{Inhibition}=\left({\text{Abs}}_{\text{control}}-{\text{Abs}}_{\text{extract}}\right)/{\text{Abs}}_{\text{control}}\times 100 \end{array}$$

### 2.6. Experimental Animals and their Maintenance

Twenty-five adult male (body weight 150-200 g, age 6-7 weeks) Wistar albino rats were purchased from BCSIR, Chittagong. The animals were randomly grouped into normal control (NC, animals received no treatment), diabetic control (DC, streptozotocin-induced and received no treatment), and treatment group (CMJE50, CMJE100, and CMJE200 mg/kg bw). The animals were individually housed in a polycarbonated cage bedded with wood husk at a temperature around 22 ± 2°C and humidity 55-60% with a 12 h light-dark cycle. All animals were supplied with a commercial pellet diet for the entire intervention period. All animal experimentations were carried out according to the guideline of the Institutional Animal Ethics Committee (EACUBS2018-4).

#### 2.6.1. Acute Oral Toxicity Test

The acute oral toxicity test was performed using standard laboratory conditions according to the “Organization for Environmental Control Development” guidelines (OECD: Guidelines 420; fixed-dose method). The allocated animals (*n* = 6) were administered a single oral dose (500 to 2000 mg/kg, body weight) of the test extract (CMJE). Before the administration of the extract, rats were fasted overnight, and food was also delayed between 3 and 4 h. After administration, food was withheld for the next 3-4 h. Experimental animals were observed individually during the first 30 minutes after dosing, periodically for the first 24 minutes (special attention for the first 4 h), with particular monitoring for possible unusual responses including allergic syndromes (itching, swelling, skin, and rash), behavioral changes, and mortality over the next 72 h. The median therapeutic effective dose was intervened as one-tenth of the median lethal dose (LD_50_ > 5.0 g/kg) [23].

#### 2.6.2. Induction of Diabetes Using Streptozotocin

Diabetes induction was accomplished with slightly modifying of protocol addressed by Al-Araby et al. [22]. Briefly, the animals were randomly divided into control and treatment groups comprising of 5 animals in each group. The normal control group (NC) received vehicle only. Diabetic control (DC) was left untreated, and the treatment groups were administered with three different doses (CMJE50, CMJE100, and CMJE200 mg/kg bw) of coconut mesocarp juice extracts. All animals except those of normal control (NC) were fed with 10% fructose solutions before one week of intraperitoneal injection of streptozotocin (50 mg/kg bw dissolved in 0.1 M citrate buffer, pH 4.5) [24] to induce diabetes which is confirmed with the fasting blood glucose level ≥ 16 mmol/L after one week of injection (measured by glucometer, Accu-Chek, USA). Once the animals were confirmed diabetic after STZ injection, each animal of CMJE50 group has been treated by the CMJE extract at the dose of 50 mg/kg bw once daily; each animal of the CMJE100 group has been treated with the CMJE extract at the dose of 100 mg/kg bw once daily, and each animal of CMJE200 group has been treated with CMJE extract at the dose of 200 mg/kg bw once daily. The treatment was continued for three weeks.

#### 2.6.3. Determination of Body Weight, Blood Glucose, and Oral Glucose Tolerance (OGT)

Weekly body weights and blood glucose levels of animals were measured and recorded. Blood glucose was measured by tail prick method using a glucometer as stated above. The glucose tolerance capacity of each animal was measured by the oral glucose tolerance test (OGTT) at the 3rd week of the intervention. Animals were administered a single dose of oral glucose solution (2 g/kg body weight) and blood glucose levels were measured at 0 (just before glucose ingestion), 30, 60, 90, and 120 min after the glucose dose.

#### 2.6.4. Animal's Blood and Organ Collection

After 4 weeks of intervention, animals were sacrificed, their blood being collected in heparinized test tubes as well as their kidney and pancreas. Blood samples were centrifuged at 3000 rpm for 15 min at 25-37°C to separate serum which was further analyzed for hepatic enzymes (alanine aminotransferase, aspartate aminotransferase), insulin, lipid profile, uric acid, and creatinine. The serum glucose level (mmol/L) was determined using the glucose test kit based on the glucose oxidase method as described [25]. The pancreas and livers were washed with 0.9% NaCl solution, wiped with tissue paper, and weighed to be preserved in 10% buffered formalin. The kidney and the pancreas were used for histopathological investigations. The homeostatic model assessment (HOMA-IR and HOMA-b) was estimated using serum insulin levels measured at the end of the experiment using the following expression:

(7) $$\begin{array}{c} \text{HOMA}‐\text{IR}=\frac{\text{Serum insulin }\left(\text{U}/\text{L}\right)\times \text{Blood glucose}\left(\text{mg}/\text{dL}\right)\;}{22.5\;},\\ \text{HOMA}-\beta -\text{cell function}=\frac{20\times \text{Serum insulin in U}/\text{L}}{\text{Blood glucose in mg}/\text{dL}-3.5}. \end{array}$$

#### 2.6.5. Histopathological Analyses

The pancreas and the kidney tissues were fixed with a buffered formalin solution for 48 h, dehydrated by passing through graded series of alcohol, and embedded in paraffin blocks [22]. The embedded tissues were sectioned at 5 *μ*m using a semiautomated rotator microtome (Biobase Bk-2258, Laboratory Manual Microtome, China). The tissue sections were then mounted on glass slides using an incubator at 60-70°C for 30 min. Afterwards, the tissue sections were deparafinized with xylene and rehydrated by using different graded ethanol dilutions (100%, 90%, and 70%). The sections were stained with hematoxylin and eosin (H&E). All slides of kidney and pancreas were examined under the Olympus BX51 Microscope (Olympus Corporation, Tokyo, Japan), and the histopathological images were taken with the help of the Olympus DP20 system under a magnification of ×200.

### 2.7. Pharmacology-Based Analysis of Thymine, Catechol, and 5-Hydroxymethylfurfural: Antidiabetic Nephropathy Ingredients

#### 2.7.1. Bioactive Compound-Target Protein Network Construction

On the basis of the network pharmacology-based prediction, STITCH 5 (http://stitch.embl.de/, ver.5.0) was used to identify target proteins related to the identified bioactive phytochemicals [25]. The software calculates a score for each pair of proteins-phytochemicals interactions. The SMILES structure of bioactive compounds (thymine, catechol, 5-hydroxymethylfurfural, 2,3-dihydro-3,5-dihydroxy-6-methyl-4H-pyran-4-one, 2H-pyran-2-methanol, 2-hydroxy-4-methyl-benzaldehyde, and 2-methylbutanoic anhydride) was put into STITCH 5 singly to match their potential targets, with the organism selected as “Homo sapiens,” the medium required interaction score being ≥0.4. We predicted a total of 84 target proteins with a medium confidence score for thymine, catechol, and 5-hydroxymethylfurfural, which were identified. The compound targets having no relationship with the compound-proteins interactions were not considered for further analysis.

#### 2.7.2. Construction of the Protein-Protein Interaction (PPI) Network of the Predicted Genes

A PPI network of the predicted genes was constructed by a search tool for the retrieval of interacting genes (STRING) database (https://string-db.org/cgi/input.pl; STRING-DB v11.0) [26]. The rank of the target proteins based on the degree of interactions in the PPI network was identified using the Cytoscape plugin cytoHubba [27]. The obtained protein interaction data of 84 target proteins were imported into the Cytoscape 3.6.1 software to construct a PPI network [28].

#### 2.7.3. Pathway Enrichment Analyses of the Target Proteins by Gene Ontology (GO) and Kyoto Encyclopedia of Genes and Genomes (KEGG)

To investigate the role of target proteins which interact with the selected phytoconstituents in gene function and signaling pathway, the Database for Annotation, Visualization, and Integrated Discovery (DAVID, https://david.ncifcrf.gov/) v6.8 was employed [29]. The KEGG [30] pathways significantly associated with the predicted genes were identified. We analyzed the Gene Ontology (GO) function and KEGG pathway enrichment of proteins (for 75 target proteins) involved in the PPI network. A brief description was made for target proteins involved in the cellular components (CC), molecular function (MF), biological process (BP), and the KEGG pathways. The adjusted *P* value < 0.05, calculated by the Benjamini–Hochberg method [31], was considered significant.

#### 2.7.4. Differential Expression of Hub Targets in the Diabetic Nephropathy Cohort

The gene expression profiling dataset GSE30122 (https://www.ncbi.nlm.nih.gov/geo/query/acc.cgi?acc=GSE30122) for diabetic nephropathy was downloaded from the NCBI gene expression omnibus (GEO) database (https://www.ncbi.nlm.nih.gov/geo/) [32]. This dataset contains tubules of 10 patients and 24 controls. An interactive web tool GEO2R (http://www.ncbi.nlm.nih.gov/geo/geo2r) was applied to screen the differential expression. The GEO2R tool used the GEOquery and limma R packages from the Bioconductor project (http://www.bioconductor.org/). To find out significant levels, the thresholds of *P* value < 0.05 and ∣FC |  (fold change) > 0.50 were ensured.

### 2.8. Statistical Analysis

All the data are presented as a mean ± SD. The data were analyzed by one-way ANOVA (analysis of variance) using the SPSS (Statistical Package for Social Science) software (version 20.0, IBM Corporation, NY) followed by Tukey's post hoc tests. The values at *P* < 0.05 were considered significantly different.

## 3. Results

### 3.1. Total Flavonoid (TFC), Total Phenolic (TPC), Lycopene, and Beta-Carotene Contents of CMJE

TFC was estimated by the standard rutin curve (*y* = 2.497*x* + 0.307, *R*^2^ = 0.979) and expressed as rutin equivalents per gram of the plant extract. The TFC of the sample was found to be 80.0 mg rutin/g while the total phenolic content using the Folin-Ciocalteu reagent method was found to be 102.0 mg GAE/g. The beta-carotene content of the extract was found to be 0.057056 mg/g, and the lycopene content was 0.0300688 mg/g. The data are presented in Table 1.

### 3.2. Antioxidative Capacity and *α*-Amylase Inhibitory Action of CMJE

**Figure 1:**
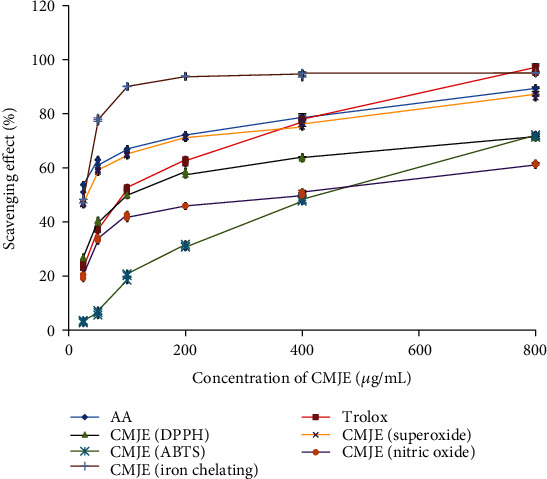
Effect of CMJE on scavenging capacities in DPPH (2,2-diphenyl 1-picrylhydrazyl) radical scavenging, SO (superoxide) scavenging, ABTS (2,2′-azino-bis(3-ethylbenzothiazoline-6-sulfonic acid)) radical scavenging, NO (nitric oxide) scavenging, and IC (iron-chelating) assays. All values were presented as means ± SD (triplicate). Data were analyzed by one-way ANOVA (analysis of variance) using the SPSS (Statistical Package for Social Science) software followed by Tukey's post hoc test.

Antioxidative capacities were measured by the DPPH scavenging method, the superoxide scavenging method, the nitric oxide scavenging method, and the iron-chelating method. The reduction of DPPH in the scavenging assay was reflected through the decrease of absorbance. The IC_90_ values of the sample (CMJE) and the standard (ascorbic acid) were found to be 123.02 ± 6.42 and 16.21 ± 2.34 *μ*g/mL, respectively. The CMJE displayed the IC_50_ value 27.85 ± 1.32 *μ*g/mL in the superoxide scavenging assay and 284.40 ± 5.05 *μ*g/mL in the nitric oxide scavenging assays. The iron-chelating capacity in terms of IC_50_ was found to be 245.47 ± 4.34 *μ*g/mL. ABTS assays showed a dose-dependent radical scavenging capacity of CMJE. The results showed the inhibition concentrations (IC_50_) 386.36 ± 1.22 *μ*g/mL for CMJE and 92.07 ± 3.21 *μ*g/mL for the standard Trolox. The comparative scavenging effect data are presented in Figure 1, and IC_50_ values are summarized in Table 2.

**Figure 2:**
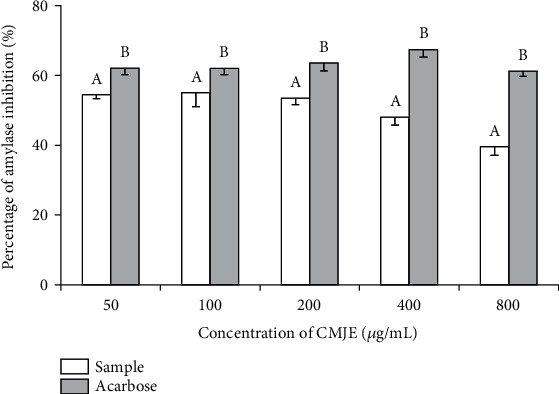
Effects of CMJE on the *α*-amylase inhibitory activity. Acarbose was used as the reference standard. Data are presented as means ± SD (triplicate). All data were analyzed by one-way ANOVA (analysis of variance) using the statistical software SPSS (Statistical Package for Social Science, version 20.0) followed by Tukey's post hoc test. Superscript letters (a, b) over the graphical bars indicate the statistical difference between inhibitory effect of CMJE and acarbose.

The *α*-amylase inhibitory effect of CMJE is presented in Figure 2. Acarbose, an antidiabetic *α*-amylase inhibitory drug, was used as a reference standard for this assay. The *α*-amylase inhibitory activity of CMJE was significantly (*P* < 0.05) lower at each concentration of acarbose. The highest inhibition for CMJE was achieved at the concentration of 100 *μ*g/mL.

### 3.3. Effects of CMJE on Blood Glucose, Glucose Tolerance, and Glucose Homeostasis

**Figure 3:**
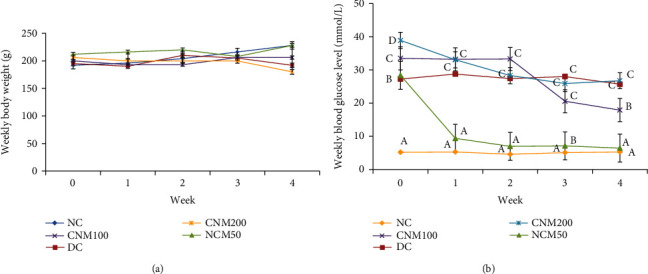
Effects of CMJE extracts on body weight (a) and weekly blood glucose levels (b) of treated animals. Data are expressed as means ± SD (*n* = 6). All data were analyzed by one-way ANOVA (analysis of variance). Significance was confirmed at *P* < 0.05. Alphabets (a–c) over the line graphs indicate the statistical differences among the groups.

CMJE was found nontoxic in the acute toxicity study. Data regarding the effect of CMJE on animals' body weight and blood glucose levels are displayed in Figure 3. The body weight of the animals was not found to vary statistically among the treatment groups, but the weight of the CMJE50 group was close to that of the normal control (NC). Data reflected that the CMJE50 group has the best glucose-lowering effect, which was statistically significant (*P* < 0.05) with the DC group.

**Figure 4:**
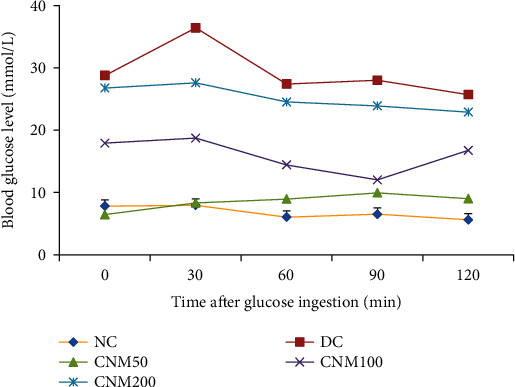
Effects of CMJE on oral glucose tolerance (OGT) at the third week of intervention. Data are expressed as means ± SD (*n* = 6). All data were analyzed by one-way ANOVA (analysis of variance) using the statistical software SPSS (Statistical Package for Social Science, version 20.0) followed by Tukey's post hoc test. Data significance was confirmed at *P* ≤ 0.05.

Glucose tolerances were assessed by the oral glucose tolerance test (OGTT) at the third week of the treatment period. Acquired data are presented in Figure 4. The glucose tolerance of the DC group was significantly (*P* < 0.05) lower than that of the other groups. However, the CMJE50 group showed the highest tolerance of glucose load than the other group, which is consistent with the other parameters achieved by this group. Effects of CMJE extracts on the glucose homeostatic status are summarized in Table 3.

### 3.4. Effects of CMJE on Pancreas and Kidney Weights

Weights of the pancreas and kidney of treated animals are listed in Table 4. Among the doses, CMJE100 in case of pancreatic recovery and CMJE50 in case of kidney recovery were found to be effective. The weight of kidneys was found to be remarkably recovered by CMJE50 while the weight of the pancreas was found to be better ameliorated by CMJE100.

### 3.5. Effects of CMJE on ALT, AST, Uric Acid, and Creatinine

Changes in ALT, AST, uric acid, and creatinine levels at the end of the intervention are presented in Table 5. The ALT levels of the different groups were not found to significantly (*P* < 0.05) differ from the NC group although the ALT level of the DC group was somehow lower than that of all other groups as well as that of the NC group. The AST level of CMJE50 and CMJE100 group is lower than that of the DC group. The uric acid level of all treated groups is almost similar to that of the NC group. Lower creatinine levels for all the treatments than DC were marked in the result.

### 3.6. Effects of CMJE on Serum Total Cholesterol and Triglyceride Levels

**Figure 5:**
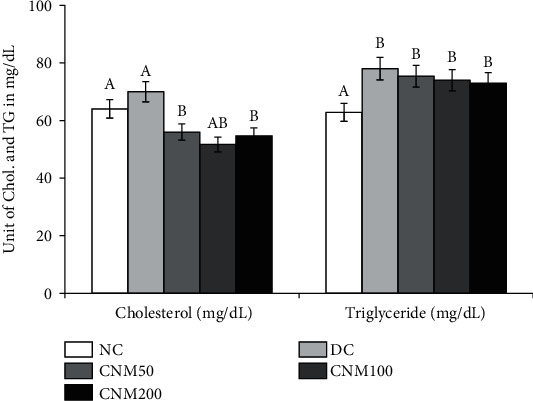
Effects of CMJE on serum cholesterol and triglyceride levels of treated animals. Data are expressed as means ± SD (*n* = 6). All data were analyzed by one-way ANOVA (analysis of variance) using the statistical software SPSS (Statistical Package for Social Science, version 20.0) followed by Tukey's post hoc test for significance at *P* ≤ 0.05. Superscript letters (a, b) on the bar graph represent the values that are significantly different compared to each other at least at the intervention period.

Both serum total cholesterol and triglyceride levels for the treatment groups were consistently lower than those of the DC group while CMJE100 was found to reduce the total cholesterol more than other doses and CMJE200 showed better effects than other doses to normalize triglyceride levels. Effects of CMJE extracts on serum total cholesterol and triglyceride are shown in Figure 5.

### 3.7. Effects of CMJE on Tissue Architecture

**Figure 6:**
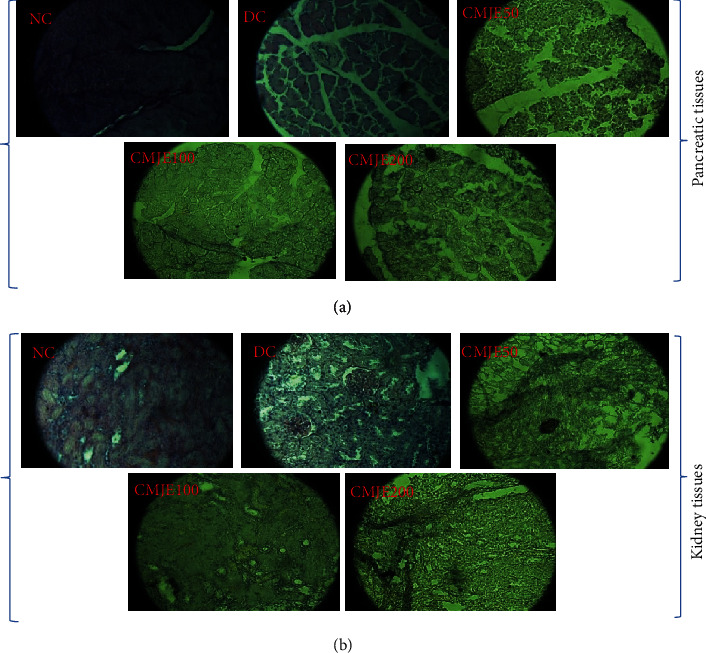
Histopathological examination by hematoxylin and eosin staining of pancreatic (a) and kidney (b) tissues after the intervention (microscopic resolution: 10 × 40). Light microscopies of pancreatic sections stained with PAS and counterstained with hematoxylin are shown. NC, DC, CMJE50, CMJE100, and CMJE200 stand for normal control (diabetic control, coconut mesocarp juice extract 50 mg/kg bw, coconut mesocarp juice extract 100 mg/kg bw, and coconut mesocarp juice extract 200 mg/kg bw).

A summary of the changes observed in the different CMJE-treatment groups is presented in Table 6. The pancreas and the kidney tissue architectures of the treated animals are presented in Figure 6. STZ induction was reflected through the decreased size of the islets of Langerhans and tissue degeneration in the pancreas of the DC group (Figure 6(a)). On the contrary, the other animal tissues were less degenerated resembling the animals' NC group. The kidney cells were remarkably recovered by CMJE50 (Figure 6(b)) while the two other treatments failed to display the same action.

### 3.8. GC-MS Compound Characterization from the CMJE

**Figure 7:**
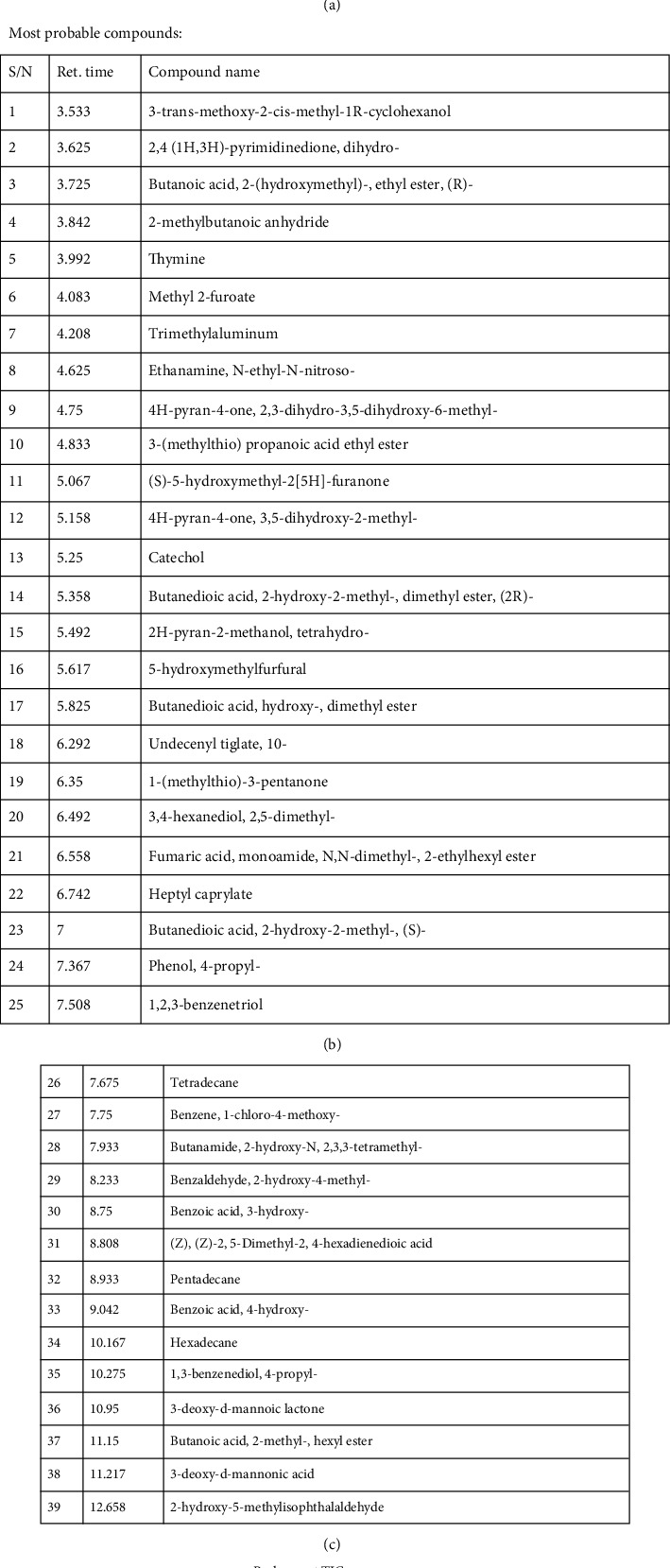
GC-MS spectra of CMJE obtained from the mass spectrometer-electron impact ionization (EI) method (GC-MS TQ 8040, Shimadzu Corporation, Kyoto, Japan) coupled with a gas chromatograph (GC-17A, Shimadzu Corporation, Kyoto, Japan). A fused silica capillary column with inlet temperature 260°C and oven temperature 70°C (0 min) was programmed. The mass range was set in the range of 50-550 m/z.

The GC-MS spectra of the CMJE extracts are shown in Figure 7. Catechol, thymine, 2,3-dihydro-3,5-dihydroxy-6-methyl-4H-pyran-4-one, 5-hydroxymethylfurfural, and 3-(methylthio)propanoic acid ethyl ester are noted as the constituents displaying the highest occurrence. The peak areas and occurrence of the compounds are summarized in Table 7.

### 3.9. Analyses of the Interactions between Active Ingredients of the Extracts and Target Proteins

To evaluate the antidiabetic activities of the constituents of the extracts, it is essential to scrutinize the target proteins on which these compounds act. We found that only thymine, catechol, and 5-hydroxymethylfurfural interacted with 84 target proteins. Based on the compound-protein targets relationships, our results indicate that these three compounds play substantial biological and physiological activities.

### 3.10. Construction and Analysis of the Target Proteins PPI Network

**Figure 8:**
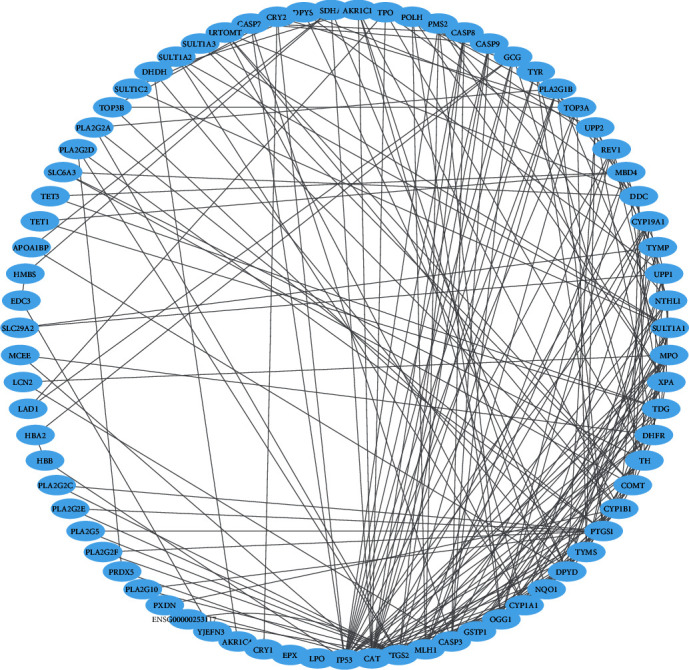
The protein-protein interaction (PPI) network of the 75 target proteins.

The PPI network analysis plays a substantial role in studying molecular processes, and abnormal PPI is at the basis of many pathological processes [33]. Using the STRING2 database, all target proteins (84) were mapped into the PPI network. Interestingly, we found that 75 target proteins were involved in PPI which have 235 edges, and an average node degree 5.88 with a PPI enrichment *P* value of less than 1.0 × 10^−16^. In this PPI network, the larger the node degree, the stronger relationship between the proteins corresponding to the node, suggesting that the target proteins plays a key role in the whole interaction network. Only nine target proteins were not included in PPI. We only got one subnetwork in PPI and this subnetwork included 75 target proteins. All target proteins with interactions with other proteins are illustrated in Figure 8. Cytoscape 3.6.1 was used to analyze the interactions among the top 15 hub target proteins (degree of interaction with no less than 10). The hub target proteins are TP53, CASP3, COMT, CYP1B1, DPYD, NQO1, PTGS1, PTGS2, CAT, OGG1, GSTP1, MLH1, CYP1A1, TYMS, and TH.

### 3.11. Gene Ontology Analyses of the Interacting Target Proteins

The GO enrichment analysis of the interacting target proteins (total 75 involved in PPI) which act with compounds of the CMJE extracts was performed by DAVID (https://david.ncifcrf.gov/). The significantly enriched terms in CC, BP, and MF categories were selected according to the Benjamini–Hochberg-corrected *P* value < 0.05. A total of 43 significant BP was listed in Table 8. In addition, two significant CC were also identified, as illustrated in Table 8.

### 3.12. Target Proteins Are Associated with the Enrichment of the KEGG Pathways

To further elucidate the relationship between target proteins and pathways, we identified 20 KEGG pathways significantly associated with the target proteins (Table 9), and they were found to be involved in secretion (pancreatic secretion), metabolism, and cellular signaling.

### 3.13. Validation of Hub Target Proteins in Diabetic Nephropathy Cohort (GSE30122)

**Figure 9:**
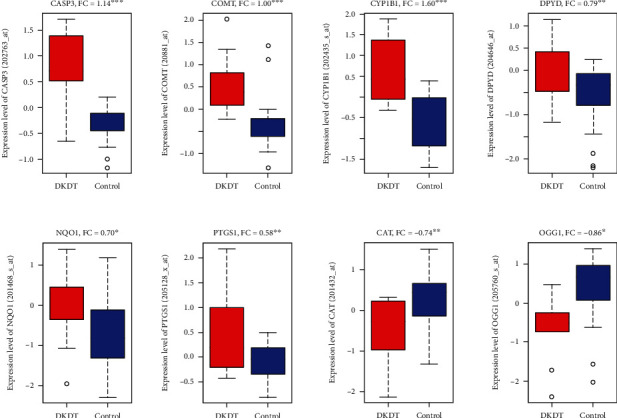
Dysregulation of hub targets (mRNA expression levels) in diabetic human kidney tubuli, when compared with control tubuli. FC: fold change, DKDT: diabetic kidney disease tubuli, control: control tubuli, ^∗^*P* < 0.05, ^∗∗^*P* < 0.01, and ^∗∗∗^*P* < 0.001.

We screened all 15 hub proteins in independent tubulointerstitial tissues of diabetic kidney disease. Interestingly, we found that 8 targets are dysregulated in this cohort. Among them, CAT and OGG1 are downregulated, and CASP3, COMT, CYP1B1, DPYD, NQO1, and PTGS1 are upregulated in diabetic kidney disease (DKD) tubuli (Figure 9).

## 4. Discussion

Coconut mesocarp juice extract (CMJE) was comprehensively studied for its antidiabetic effects which have been evaluated in the light of CMJE's phytoconstituent status and antioxidative potential. It is believed that oxidative stress plays an important role in the development of vascular complications in diabetes particularly type 2 diabetes [34]. Free radical formation in diabetes by nonenzymatic glycation of proteins, glucose oxidation, and increased lipid peroxidation leads to damage of enzymes and cellular machinery and increases insulin resistance [35]. Free radicals, therefore, through their aforesaid abilities play a major role in damaging lipids, proteins, and DNA [36] in the onset of diabetic complication. Additionally, the elevation of reactive oxygen species (ROS) decreases the production of biological antioxidative enzymes such as catalase, superoxide dismutase (SOD), and glutathione peroxidase (GSH-Px) [37]. Variations in the levels of these enzymes render the tissues susceptible to oxidative stress leading to the development of diabetic complications [38]. Our data are strongly supportive for the antidiabetic action of CMJE extracts because of their high total phenolic content, total flavonoid content, and carotenoid and lycopene contents. Similar researches on the antidiabetic actions of plant phenolics especially flavonoids, triterpenoids, and saponins have been published recently [39, 40]. Phenolic compounds have attracted tremendous interest due to their outstanding free radical scavenging capacity which is measured by both in vivo and in vitro methods; some of in vitro methods, such as DPPH, ABTS, superoxide, NO, iron-chelating assays, were opted for our CMJE samples. Promising free radical scavenging effects of CMJE noted with our extracts imply their potential to be reflected in cellular systems for reducing oxidative stress [41, 42], a pivotal factor for diabetes and diabetes-related diseases, leading to attenuate diabetic complications [43]. The use of CMJE extracts, therefore, might have a highly prospective use as antioxidative food supplements in diabetes treatment [44].

One of the methods used to treat diabetes mellitus is the inhibition of carbohydrate-digesting enzymes such as *α*-amylase and *α*-glucosidase upon gastrointestinal glucose absorption [21]. In this study, the effect of the mesocarp part of coconut fruit was evaluated on the activity of *α*-amylase, which could constitute a basis for searching alternative drugs from CMJE extracts. This finding is in line with previous reports which showed that promising inhibition of pancreatic *α*-amylase could lead to the fermentation of undigested carbohydrates by bacteria in an irregular way in the colon, such that a mild activity of *α*-amylase inhibition is desirable [44]. This suggests that the three interactive components characterized here in the CMJE extracts may compete with the substrate for binding to the active site of the enzyme thereby preventing the breaking down of oligosaccharides to disaccharides.

In a study using the animals, body weights were found to be higher than that in the diabetic control, which is literally the sign of ameliorating the trend of diabetes. Weekly blood glucose and oral glucose tolerance have tremendously been favored by the lowest dose of CMJE50, which is seemingly a very remarkable and promising feature for the further use of CMJE extracts as drug molecules. Probably, the recovery of the pancreas in the STZ-induced rats was the highest with the administration of the coconut aqueous extract, as a consequence of the restoration of glucose levels reflected by the higher pancreatic weights of CMJE-treated groups compared to diabetic rats. The pancreas weight loss observed in the DC group could be related to both the destruction and the disappearance of pancreatic islet cells as well as the selective disruption of insulin-producing cells [45].

The increase in aminotransferase levels recorded in our study may be linked to cellular damage in the liver caused by STZ-induced diabetes. Lowering of total cholesterol and triglycerides could be ascribed as an effective role of CMJE extracts in controlling diabetic dyslipidemia, a harbinger of future diabetes, characterized by increased triglycerides, and postprandial lipemia [46]. Improvement of the tissue architecture of the pancreas and the kidney could be ascertained through the absence of tubular epithelial cell necrosis and the presence of degenerated cells, although the lowest CMJE dose was more effective than the others. This could be explained by the receptor occupancy of respective cells with the lower CMJE doses. Additionally, low CMJE doses proportionally cause smaller digestive disturbance and are responsible for higher drug absorption. Low digestive disturbance thus leads to higher insulin sensitivity to control the blood glucose level to a higher extent [47]. The Hormesis effect may be attributed to trigger the receptors with lower doses of treatment.

The effects of CMJE extracts described above could be linked with the presence of a few polyphenolic compounds characterized by GC-MS, such as catechol, 4-hydroxybenzoic acid, 1-chloro-4-methoxy-benzene, methyl 2-furoate, thymine, 4-propyl-phenol, 2,3-dihydro-3,5-dihydroxy-6-methyl-4H-pyran-4-one, 5-hydroxymethylfurfural, and 2-hydroxy-5-methylisophthalaldehyde, which are well known as antioxidant and antidiabetic constituents. The aforesaid statement has been confirmed by network pharmacological bioinformatics tools using the PPI network and gene ontology analyses.

The PPI network plays substantial roles in studying molecular processes, and abnormal PPI is at the basis of many pathological processes [48]. From the PPI of targeted proteins, we identified hub nodes which are markedly associated with diabetes. For example, ABCA1 protein with 10 degrees of interaction plays an inevitable role in regulating lipid metabolism, and defect in this gene disrupts lipid transport of HDL-cholesterol associated with the development of T2DM [49]. TNF-alpha concentration is also linked with peripheral insulin resistance and elevated plasma glucose as well as insulin levels before the onset of type 2 diabetes [50]. It was observed that a CXCL8 antagonist ameliorates diabetic nephropathy in diabetic male mice and attenuates high glucose-induced mesangial injury [51]. Caspase-3 promotes diabetic kidney disease [52]. The *HMGCR* gene, in population studies, was also found to be associated with bodyweight gain and a higher risk of type 2 diabetes [53]. Interleukin-10 (IL10), an anti-inflammatory cytokine, is supposed to play a type 2 diabetes (T2D) protective role [54]. Figure 9 shows that the immunological target proteins including IL10, CXCL8, and TNF are located in the PPI networks with a top degree of interaction, indicating that this PPI network is associated with immunological activities. It was stated that humans' chemokines have been associated with, or implicated in, the pathogenesis of type 1 diabetes [55]. Altogether, these compound-proteins interactions may be associated with the regulation of diabetes pathophysiology.

In addition, we identified the GO and pathway enrichment of target proteins. The GO analysis indicated that the target proteins may bind with plasma membranes, chromosomes, chromatin, regulatory regions of nucleic acids, or/and cellular receptors of cells for mediating metabolic, immunological processes, signaling, and/or other activities, so as to exert signaling and antidiabetic potentials [56]. Pathway analysis revealed some pathways being also associated with diabetes. Retinol metabolism is the most significantly enriched pathway (FDR < 8.6 × 10^−6^). Retinoids and retinoid-related proteins are associated with signaling molecules linking obesity with the development of type 2 diabetes and in the pancreatic *β*-cell biology/insulin secretion [57]. Additionally, *CAT* and *SOD1* genes/proteins are assumed to highly influence the control of the PPI network because these are also known as antioxidative enzymes having a very potent role in diabetes and diabetes-related complications including diabetic nephropathy [57]. Apart from these observations, the 15 hub genes are targeted in independent diabetic kidney disease (DKD). Interestingly, dysregulation of eight of those genes in this cohort complies with the fact that *CAT* and *OGG1* are downregulated and *CASP3*, *COMT*, *CYP1B1*, *DPYD*, *NQO1*, and *PTGS1* are upregulated in DKD, suggesting that the target compounds are clearly interacting with potential genes associated with diabetic nephropathy [58]. Moreover, metabolic, oxidative, oxidant detoxification, and inflammatory stresses are common features in diabetic nephropathy [59]. In streptozotocin-induced diabetic rats, modulation of xenobiotic metabolism and oxidative stress in various tissues may be related to altered metabolism [60]. Peroxidase activity, a top molecular function, is increased in advanced diabetic nephropathy [61]. Phospholipase A2 activity is a risk factor in diabetic nephropathy [62]. Altogether, these GO are clearly associated with the dysregulation of diabetic kidney disease (DKD).

The KEGG pathways were mainly involved in secretion (pancreatic secretion), metabolism, and cellular signaling. In type 2 diabetes, alpha-linolenic acid has effects on the control of the glycemic index [63]. Yan et al. revealed that antidiabetic agents are associated with arachidonic acid metabolism, glycerophospholipid metabolism, tryptophan metabolism, and tyrosine metabolism [64]. Another pathway, apoptosis, is also critically associated with diabetes [65]. Collectively, these enriched pathways are associated with the DKD. In a diabetic nephropathy cohort study, *CAT* is a downregulated gene which may be associated with the regulation of kidney functions in diabetes [66]. The gene *CASP3* promotes the DKD through secondary necrosis [53]. Another upregulated gene, *CYP1B1*, is also associated with the damage and dysfunction of renal functions in mice. Increased expression levels of *PTGS1* are associated with the progression of diabetic nephropathy [67] suggesting that these are the potential genes, interacting with our target compounds, which are dysregulated in diabetic kidney diseases.

## 5. Conclusions

Our investigations revealed that the CMJE partially restored biochemical markers especially ALT, AST, creatinine, uric acid, and lipid profiles and improved glucose homeostasis as well as tissue architecture. The target compounds of CMJE downregulated *CAT* and *OGG1* genes and upregulated *CASP3*, *COMT*, *CYP1B1*, *DPYD*, *NQO1*, and *PTGS1* genes implying to potentiate their antioxidative actions to protect the dysregulation of the pancreas and the kidney of STZ-diabetic animals. Therefore, the coconut mesocarp juice extract is suggested to produce antidiabetic actions in induced animal models. A further study of in vivo antioxidative effects both in enzymatic and nonenzymatic systems might confirm the use of CMJE as alternative therapeutic in diabetic complications.

## Figures and Tables

**Table 1:** Total phenolic, total flavonoid, lycopene, and carotenoid contents of CMJE.

Phytochemical index	Quantity
Total flavonoid	80.0 mg rutin/g
Total phenolic content	102.0 mg GAE/g
Lycopene	0.031 mg/g
Total carotenoids	0.058 mg/g

**Table 2:** Comparative IC_50_ values achieved by CMJE in different antioxidative models.

Antioxidative models	Test sample	Reference standard	Inhibition concentration (IC_50_, *μ*g/mL)	
			CMJE	Reference standard
DPPH scavenging assay	CMJE	Ascorbic acid	123.02 ± 6.42	
Superoxide scavenging assay	CMJE	Ascorbic acid	27.85 ± 1.32	16.21 ± 2.34
Nitric oxide assay	CMJE	Ascorbic acid	284.40 ± 5.05	
Iron-chelating assay	CMJE	Ascorbic acid	245.47 ± 4.34	
ABTS assay	CMJE	Trolox	386.36 ± 1.22	92.07 ± 3.21

**Table 3:** Effects of CMJE on the glucose homeostatic status (HOMA-IR and HOMA-*β*).

Treatment groups	HOMA-IR (mIU/L)	HOMA-*β* (%)
NC	0.017	0.460
DC	0.116	0.036
CMJE50	0.052	0.240
CMJE100	0.155	0.045
CMJE200	0.111	0.082

HOMA-IR stands for homeostatic model assessment for insulin resistance, and HOMA-*β* represents the pancreatic beta cell function (%).

**Table 4:** Effects of CMJE on the relative weight of the pancreas and kidney of treated animals.

Tissue weight	Pancreas weight (g)	Kidney weight (g)
NC	0.569 ± 0.105^a^	1.794 ± 0.106^a^
DC	0.304 ± 0.047^b^	1.738 ± 0.093^a^
CMJE50	0.215 ± 0.044^c^	1.772 ± 0.127^a^
CMJE100	0.320 ± 0.050^b^	1.690 ± 0.031^a^
CMJE200	0.215 ± 0.035^c^	1.663 ± 0.199^a^

Data are expressed as means ± SD (*n* = 6). All data were analyzed by one-way ANOVA (analysis of variance) using the statistical software SPSS (Statistical Package for Social Science, version 20.0) followed by Tukey's post hoc test. Data significance was confirmed at *P* ≤ 0.05. The superscript alphabets (a–c) in the table denote the reciprocal significance between and among the groups.

**Table 5:** Effects of CMJE on serum ALT, AST, uric acid and creatinine levels.

Treatment groups	ALT (U/L)	AST (U/L)	Uric acid (mg/dL)	Creatinine (mg/dL)
NC	71.60 ± 2.40^a^	4.80 ± 1.76^a^	6.60 ± 2.40^a^	0.49
DC	33.75 ± 2.50^b^	7.62 ± 2.00^b^	12.00 ± 2.80^b^	1.23
CMJE50	73.5 0 ± 7.20^a^	6.00 ± 1.2 0^c^	6.30 ± 0.72^a^	0.71
CMJE100	80.00 ± 8.00^a^	5.17 ± 1.04^c^	6.59 ± 0.82^a^	0.77
CMJE200	79.00 ± 8.10^a^	6.50 ± 0.85^b^	7.18 ± 0.71^c^	0.68

Data are expressed as means ± SD (*n* = 6). All data were analyzed by one-way ANOVA (analysis of variance) using the statistical software SPSS (IBM Corporation, NY, version 20.0) followed by Tukey's post hoc test for significance at *P* ≤ 0.05. The significant differences among and between the groups at least in the experimental condition are represented through the superscript letters (a–c) in the table.

**Table 6:** Effect of CMJE on the tissue architectures of the pancreas and kidney.

Name of the tissues and parameters	Group				
	NC	DC	CMJE50	CMJE100	CMJE200
Pancreas					
Degenerated cells	-	+++	++	+	+
Necrotic cells	-	+++	+	+	++
Kidney					
Atrophic glomerulus and tubules	-	++	-	-	-
Eosinophilic secretion in the tubules lumen	-	-	-	-	-
Tubular epithelial cell degeneration	-	+++	-	++	++
Increased fibrous tissue	-	++	+	+	+
Hyperemic vessels in the interstitium	-	+++	+	+	+

Histopathological assessments are graded as follows: (i) (-) indicates “no abnormality.” (ii) (+) indicates “mild injury.” (iii) (++) indicates “moderate injury.” (iv) (+ + +) indicates “severe injury”.

**Table 7:** Compounds obtained from GC-MS analyses of the CMJE.

SL No.	Compound name	RT	Peak area (%)
1	3-Trans-methoxy-2-cis-methyl-1R-cyclohexanol	3.53	1.27
2	2,4(1H, 3H)-Pyrimidinedione, dihydro	3.625	1.58
3	Butanoic acid, 2-(hydroxymethyl)-ethyl ester (R)-	3.72	0.52
4	2-Methylbutanoic anhydride	3.844	3.61
5	Thymine	3.994	7.1
6	Methyl 2-furoate	4.083	1.73
7	Trimethylaluminum	4.210	0.96
8	Ethanamine, N-ethyl-N-nitroso-	4.624	1.91
9	2,3-Dihydro-3,5-dihydroxy-6-methyl-4H-pyran-4-one	4.751	12.94
10	3-(Methylthio)propanoic acid ethyl ester	4.833	7.24
11	(S)-5-Hydroxymethyl-2[5H]-furanone	5.064	0.71
12	3,5-Dihydroxy-2-methyl-4H-pyran-4-one	5.158	0.34
13	Catechol	5.253	7.20
14	Butanedioic acid, 2-hydroxy-2-methyl-, dimethyl ester, (2R)-	5.358	1.00
15	2H-pyran-2-methanol, tetrahydro-	5.494	5.20
16.	5-Hydroxymethylfurfural	5.618	14.41
17	Butanedioic acid, hydroxy-, dimethyl ester	5.82	1.71
18	Undecenyl tiglate, 10-	6.291	1.07
19	1-(Methylthio)-3-pentanone	6.353	1.86
20	3,4-Hexanediol, 2,5-dimethyl-	6.49	1.26
21	Fumaric acid, monoamide, N,N-dimethyl-, 2-ethylhexyl ester	6.561	0.78
22	Heptyl caprylate	6.741	1.57
23	Butanedioic acid, 2-hydroxy-2-methyl-, (S)-	7.002	1.08
24	Phenol, 4-propyl-	7.364	1.58
25	1,2,3-Benzenetriol	7.505	2.44
26	Tetradecane	7.674	1.20
27	Benzene, 1-chloro-4-methoxy-	7.75	0.42
28	Butanamide, 2-hydroxy-N,2,3,3-tetramethyl-	7.933	1.04
29	2-hydroxy-4-methyl-benzaldehyde	8.23	3.70
30	(Z),(Z)-2,5-Dimethyl-2,4-hexadienedioic acid	8.808	1.34
31	Pentadecane	8.933	1.50
32	Hexadecane	10.168	1.04
33	1,3-Benzenediol, 4-propyl-	10.273	0.81
34	3-Deoxy-d-mannoic lactone	10.948	2.49
35	Butanoic acid, 2-methyl-, hexyl ester	11.150	0.40
36	3-Deoxy-d-mannonic acid	11.213	0.95
37	2-Hydroxy-5-methylisophthalaldehyde	12.658	0.87

**Table 8:** Gene Ontology (GO) enrichment analysis of the interacting target proteins; 43 biological processes, 15 molecular functions, and 2 cellular components.

Category	Term	Benjamini-corrected *P* value
BP	GO:0042744, hydrogen peroxide catabolic process	5.78*E* − 12
	GO:0098869, cellular oxidant detoxification	1.08*E* − 10
	GO:0055114, oxidation-reduction process	6.90*E* − 10
	GO:0050482, arachidonic acid secretion	1.03*E* − 09
	GO:0036149, phosphatidylinositol acyl-chain remodeling	5.02*E* − 09
	GO:0036148, phosphatidylglycerol acyl-chain remodeling	9.64*E* − 09
	GO:0036150, phosphatidylserine acyl-chain remodeling	9.64*E* − 09
	GO:0032355, response to estradiol	1.53*E* − 08
	GO:0036152, phosphatidylethanolamine acyl-chain remodeling	5.14*E* − 08
	GO:0006979, response to oxidative stress	6.62*E* − 08
	GO:0036151, phosphatidylcholine acyl-chain remodeling	8.95*E* − 08
	GO:0006654, phosphatidic acid biosynthetic process	4.34*E* − 07
	GO:0006805, xenobiotic metabolic process	2.18*E* − 06
	GO:0016042, lipid catabolic process	3.67*E* − 06
	GO:0046135, pyrimidine nucleoside catabolic process	4.76*E* − 06
	GO:0006644, phospholipid metabolic process	4.76*E* − 06
	GO:0045471, response to ethanol	1.29*E* − 05
	GO:0007568, aging	1.91*E* − 05
	GO:0008202, steroid metabolic process	3.89*E* − 05
	GO:0045008, depyrimidination	1.48*E* − 04
	GO:0042493, response to drug	1.82*E* − 04
	GO:0032496, response to lipopolysaccharide	1.97*E* − 04
	GO:0006584, catecholamine metabolic process	1.91*E* − 04
	GO:0046677, response to antibiotic	2.95*E* − 04
	GO:0051923, sulfation	3.42*E* − 04
	GO:0006284, base-excision repair	3.92*E* − 04
	GO:0050427, 3′-phosphoadenosine 5′-phosphosulfate metabolic process	8.60*E* − 04
	GO:0032025, response to cobalt ion	6.80*E* − 03
	GO:0009635, response to herbicide	9.14*E* − 03
	GO:0009636, response to toxic substance	1.11*E* − 02
	GO:0009308, amine metabolic process	1.13*E* − 02
	GO:0009812, flavonoid metabolic process	1.13*E* − 02
	GO:0008635, activation of cysteine-type endopeptidase activity involved in apoptotic process by cytochrome c	1.40*E* − 02
	GO:0043066, negative regulation of apoptotic process	1.42*E* − 02
	GO:0042416, dopamine biosynthetic process	1.64*E* − 02
	GO:0043525, positive regulation of neuron apoptotic process	1.63*E* − 02
	GO:0008210, estrogen metabolic process	1.89*E* − 02
	GO:0043097, pyrimidine nucleoside salvage	2.19*E* − 02
	GO:0042542, response to hydrogen peroxide	2.45*E* − 02
	GO:0071407, cellular response to organic cyclic compound	3.62*E* − 02
	GO:0097194, execution phase of apoptosis	3.61*E* − 02
	GO:0080111, DNA demethylation	3.61*E* − 02
	GO:0033189, response to vitamin A	4.45*E* − 02
CC	GO:0005829, cytosol	4.25*E* − 06
	GO:0005739, mitochondrion	1.18*E* − 04
MF	GO:0004601, peroxidase activity	5.24*E* − 12
	GO:0020037, heme binding	5.97*E* − 11
	GO:0004623, phospholipase A2 activity	3.61*E* − 09
	GO:0003684, damaged DNA binding	1.60*E* − 05
	GO:0005506, iron ion binding	1.30*E* − 05
	GO:0019825, oxygen binding	6.41*E* − 05
	GO:0004062, aryl sulfotransferase activity	5.34*E* − 04
	GO:0097153, cysteine-type endopeptidase activity involved in apoptotic process	6.06*E* − 04
	GO:0008146, sulfotransferase activity	1.35*E* − 02
	GO:0047498, calcium-dependent phospholipase A2 activity	1.48*E* − 02
	GO:0019104, DNA N-glycosylase activity	1.67*E* − 02
	GO:0030983, mismatched DNA binding	1.87*E* − 02
	GO:0016712, oxidoreductase activity, acting on paired donors, with incorporation or reduction of molecular oxygen, reduced flavin or flavoprotein as one donor, and incorporation of one atom of oxygen	3.23*E* − 02
	GO:0004497, monooxygenase activity	3.21*E* − 02
	GO:0004197, cysteine-type endopeptidase activity	3.46*E* − 02

**Table 9:** Enriched KEGG pathways significantly associated with target proteins in this study.

KEGG pathway	Benjamini-corrected *P* value
hsa00592: alpha-linolenic acid metabolism	1.96*E* − 07
hsa00590: arachidonic acid metabolism	1.81*E* − 07
hsa00591: linoleic acid metabolism	2.06*E* − 07
hsa04975: fat digestion and absorption	1.42*E* − 06
hsa00565: ether lipid metabolism	3.21*E* − 06
hsa01100: metabolic pathways	5.47*E* − 05
hsa04972: pancreatic secretion	3.39*E* − 04
hsa00564: glycerophospholipid metabolism	3.42*E* − 04
hsa05204: chemical carcinogenesis	1.10*E* − 03
hsa04270: vascular smooth muscle contraction	1.05*E* − 03
hsa03460: fanconi anemia pathway	1.23*E* − 03
hsa00140: steroid hormone biosynthesis	1.74*E* − 03
hsa00350: tyrosine metabolism	2.51*E* − 03
hsa00980: metabolism of xenobiotics by cytochrome P450	4.69*E* − 03
hsa00983: drug metabolism—other enzymes	6.29*E* − 03
hsa00240: pyrimidine metabolism	1.66*E* − 02
hsa04210: apoptosis	1.69*E* − 02
hsa03410: base-excision repair	2.19*E* − 02
hsa04014: ras signaling pathway	2.74*E* − 02
hsa00380: tryptophan metabolism	3.39*E* − 02

## Data Availability

The data used to support the findings of this study are available from the corresponding authors upon request.

## Abbreviations

CMJE: Coconut mesocarp juice extract
TFC: Total flavonoid content
TPC: Total phenolic content
SO: Superoxide scavenging effect
DPPH-1: 1-Diphenyl, 2-picrylhydrazyl free radical scavenging assay
NO: Nitric oxide scavenging effect
IrC: Iron-chelating effect
ABTS: (2,2′-azino-bis(3-ethylbenzothiazoline-6-sulfonic acid)
ALT: Alanine aminotransferase
AST: Aspartate aminotransferase
HOMA-IR: Glucose homeostasis
SPSS: Statistical Package for Social Science
TCh: Total cholesterol
TG: Triglyceride
PPI: Protein-protein interaction
GO: Gene Ontology
KEGG: Kyoto Encyclopedia of Genes and Genomes
BP: Biological process
MF: Molecular function
CC: Cellular component groups.

## Additional Points

*Institutional Review Board Statement*. Animal handling and care were ensured through the ethical guideline formed in accord with the Helsinki protocol. All animal experimentations were carried out according to the guideline of Institutional Animal Ethics Committee (EACUBS2018-4).

## Supplementary Materials

Supplementary Materials Table S1: list of compounds-targets identification. Table S2: list of targets involved in PPI with degree of interactions. Table S3: ontology (GO) enrichment analysis of the interacted target proteins. Table S4: the enriched KEGG pathways which are significantly associated with target proteins. Figure S1: a comprehensive approach to display the effect of coconut mesocarp juice extract on the streptozotocin-induced diabetes and diabetes-related complications using in vitro, in vivo and computational models.
