# Supplementary material for: The Antioxidative Role of Natural Compounds from a Green Coconut Mesocarp Undeniably Contributes to Control Diabetic Complications as Evidenced by the Associated Genes and Biochemical Indexes
Source: Oxid Med Cell Longev. 2021 Jul 27;2021:9711176. doi: 10.1155/2021/9711176 (PMC8337112; doi:10.1155/2021/9711176)
Supplement: Supplementary Materials — Table S1: list of compounds-targets identification. Table S2: list of targets involved in PPI with degree of interactions. Table S3: ontology (GO) enrichment analysis of the interacted target proteins. Table S4: the enriched KEGG pathways which are significantly associated with target proteins. Figure S1: a comprehensive approach to display the effect of coconut mesocarp juice extract on the streptozotocin-induced diabetes and diabetes-related complications using in vitro, in vivo and computational models. [file 9711176.f1.docx]

**Original article**

**Antioxidative role of natural compounds from green Coconut mesocarp undeniably contribute to control diabetic complications as evidenced by the associated genes and biochemical indexes**

Rickta Rani Das^a^, Md. Atiar Rahman^a^*, SalahuddinQader Al-Araby^a^, Md. ShahidulIslam^a^, Md. MamunurRashid^a^, NoufAbubakrBabteen^b^, Afnan M. Alnajeebi^b^, Hend Faisal H. Alharbi^c^, Philippe Jeandet^d^, Khalid JuhaniRafi^a^, Tanvir Ahmed Siddique^a^, Md. NazimUddin^e^and ZainulAmiruddinZakaria^f^*

^a^Department of Biochemistry and Molecular Biology, University of Chittagong, Chittagong-4331, Bangladesh.

^b^Department of Biochemistry, College of Science, University of Jeddah, Jeddah 80203, Saudi Arabia

^c^Department of Food Science and Human Human Nutrition, Collage of Agriculture and Veterinary Medicine, Qassim University, Saudi Arabia

^d^Department of Biology and Biochemistry, Faculty of Sciences, University of Reims, PO Box 1039, France

^e^Institute of Food Science and Technology, Bangladesh Council of Scientific and Industrial Research, Dhaka 1205, Bangladesh

^f^Department of Biomedical Science, Faculty of Medicine and Health Sciences, Universiti Putra Malaysia, UPM Serdang 43400, Selangor, Malaysia

**Running Title**: Antidiabetic potential of coconut mesocarp juice extract

***Correspondence: author**: Md. Atiar Rahman PhD, Professor, Department of Biochemistry & Molecular Biology, University of Chittagong, Chittagong-4331, Bangladesh, Tel: +88-031-2606001-10, Extension- 4334, Fax: +88-031-726310, E-mail: atiar@cu.ac.bd

**and**

***Correspondence**: ZainulAmiruddinZakaria, Professor, Department of Biomedical Science, Faculty of Medicine and Health Sciences, Universiti Putra Malaysia, UPM Serdang 43400, Selangor, Malaysia, Email: zaz@upm.edu.my

Serum collection and analyses for ALT, AST, Creatinine, Uric acid, lipid profile

Liver glycogen

Compound-target interactions

PPI Network

Network KEGGpathway

GO Ontology

Protein-protein interactions

**Reduced Oxidative stress leads to attenuate diabetic complication**

GCMS analysis


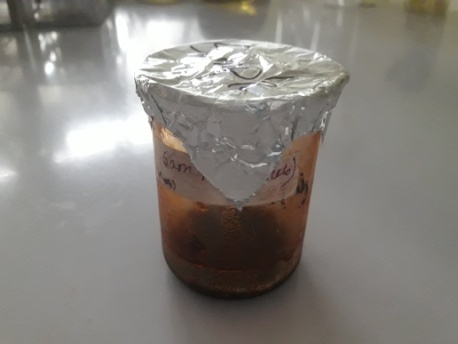


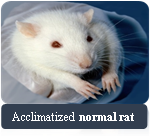

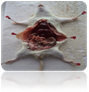

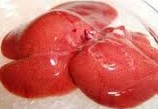

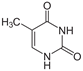

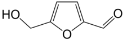

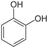

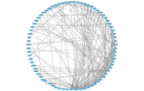

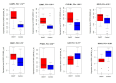


Restorationofdiabetes and diabetes related nephropathy

Oral glucose tolerance

*Cocos nucifera mesocarp extract*


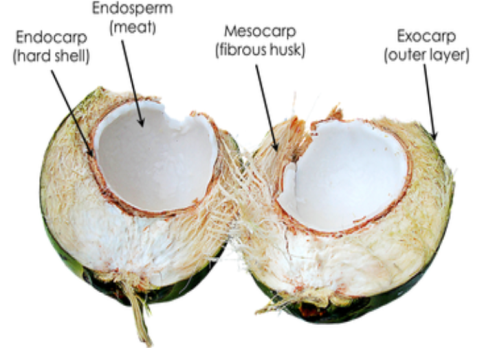


Four weeks intervention

Extract

Antioxidative effect

Α-amylase inhibitory effect

**Figure S1:** A comprehensive approach to display the effect of Coconut mesocarp juice extract on the Strepozotocin-induced diabetes and diabetes related complications using in vitro, in vivo and computational models.

| **Supplementary table S1. List of compounds-targets identification** | | |
| --- | --- | --- |
| **Catechol** | **Thymine** | **5-Hydroxymethylfurfural** |
| CASP9 | POLH | HBB |
| CASP8 | PMS2 | HBA2 |
| GSTP1 | DHFR | SULT1A1 |
| CYP1A1 | DPYD | SULT1A2 |
| TH | TYMP | SDHA |
| PLA2G10 | CRY2 |  |
| PLA2G2A | TP53 |  |
| CASP7 | XPA |  |
| PLA2G5 | UPP1 |  |
| PTGS2 | MBD4 |  |
| TYR | TDG |  |
| COMT | TOP3A |  |
| PTGS1 | DPYS |  |
| CYP1B1 | UPP2 |  |
| LRTOMT | OGG1 |  |
| DDC | SLC29A2 |  |
| TPO | MLH1 |  |
| PLA2G2F | ATAD2 |  |
| DHDH | APOA1BP |  |
| PLA2G2E | TYMS |  |
| LCN2 | YJEFN3 |  |
| AKR1C4 | ENSG00000258674 |  |
| AKR1C1 | CRY1 |  |
| PRDX5 | EDC3 |  |
| LPO | TOP3B |  |
| GCG | TET3 |  |
| PLA2G1B | REV1 |  |
| SULT1A3 | TET1 |  |
| MPO | NTHL1 |  |
| CASP3 | TGIF2 |  |
| EPX | LAD1 |  |
| SULT1A1 |  |  |
| HMBS |  |  |
| SULT1A2 |  |  |
| HBB |  |  |
| CAT |  |  |
| SULT1C2 |  |  |
| MCEE |  |  |
| PXDNL |  |  |
| PXDN |  |  |
| PLA2G2C |  |  |
| NQO1 |  |  |
| OC90 |  |  |
| ENSG00000258417 |  |  |
| PLA2G2D |  |  |
| GSTT1 |  |  |
| CYP19A1 |  |  |
| SLC6A3 |  |  |

**Supplementary table S2. List of targets involved in PPI with degree of interactions**

| Rank | Name | Score |
| --- | --- | --- |
| 1 | TP53 | 28 |
| 2 | CAT | 26 |
| 3 | PTGS2 | 23 |
| 4 | GSTP1 | 14 |
| 4 | CASP3 | 14 |
| 4 | MLH1 | 14 |
| 7 | OGG1 | 13 |
| 7 | CYP1A1 | 13 |
| 9 | PTGS1 | 12 |
| 9 | NQO1 | 12 |
| 9 | TYMS | 12 |
| 9 | DPYD | 12 |
| 9 | CYP1B1 | 12 |
| 14 | COMT | 11 |
| 15 | TH | 10 |
| 16 | MPO | 9 |
| 16 | XPA | 9 |
| 16 | TDG | 9 |
| 16 | DHFR | 9 |
| 16 | SULT1A1 | 9 |
| 21 | NTHL1 | 8 |
| 22 | CYP19A1 | 7 |
| 22 | DDC | 7 |
| 22 | TYMP | 7 |
| 22 | UPP1 | 7 |
| 26 | TYR | 6 |
| 26 | MBD4 | 6 |
| 26 | GCG | 6 |
| 26 | PLA2G1B | 6 |
| 26 | CASP8 | 6 |
| 26 | CASP9 | 6 |
| 26 | REV1 | 6 |
| 26 | TOP3A | 6 |
| 26 | UPP2 | 6 |
| 35 | TPO | 5 |
| 35 | PMS2 | 5 |
| 35 | POLH | 5 |
| 35 | AKR1C1 | 5 |
| 39 | SULT1A3 | 4 |
| 39 | LRTOMT | 4 |
| 39 | CASP7 | 4 |
| 39 | SDHA | 4 |
| 39 | CRY2 | 4 |
| 39 | SULT1A2 | 4 |
| 39 | DPYS | 4 |
| 46 | SLC6A3 | 3 |
| 46 | SULT1C2 | 3 |
| 46 | PLA2G2A | 3 |
| 46 | TOP3B | 3 |
| 46 | PLA2G2D | 3 |
| 46 | DHDH | 3 |
| 52 | LCN2 | 2 |
| 52 | MCEE | 2 |
| 52 | TET1 | 2 |
| 52 | PXDN | 2 |
| 52 | PLA2G10 | 2 |
| 52 | LAD1 | 2 |
| 52 | HMBS | 2 |
| 52 | APOA1BP | 2 |
| 52 | TET3 | 2 |
| 52 | ENSG00000253117 | 2 |
| 52 | PLA2G2F | 2 |
| 52 | HBB | 2 |
| 52 | HBA2 | 2 |
| 52 | SLC29A2 | 2 |
| 52 | PLA2G5 | 2 |
| 52 | PLA2G2E | 2 |
| 52 | PLA2G2C | 2 |
| 52 | EDC3 | 2 |
| 52 | PRDX5 | 2 |
| 71 | AKR1C4 | 1 |
| 71 | EPX | 1 |
| 71 | CRY1 | 1 |
| 71 | LPO | 1 |
| 71 | YJEFN3 | 1 |

Supplementary Table S3. Ontology (GO) enrichment analysis of the interacted target proteins

| **Category** | **Term** | **Count** | **P Value** | **Genes** | **Benjamini corrected P value** |
| --- | --- | --- | --- | --- | --- |
| **BP** | GO:0042744~hydrogen peroxide catabolic process | 9 | 8.18E-15 | LPO, PXDN, EPX, TPO, MPO, PRDX5, HBA2, CAT, HBB | 5.78E-12 |
|  | GO:0098869~cellular oxidant detoxification | 11 | 3.07E-13 | LPO, PXDN, PTGS2, EPX, PTGS1, TPO, PRDX5, HBA2, CAT, HBB, GSTP1 | 1.08E-10 |
|  | GO:0055114~oxidation-reduction process | 20 | 2.94E-12 | LPO, PXDN, CYP1B1, PTGS2, CYP1A1, TH, PTGS1, PRDX5, DHDH, SDHA, TYR, AKR1C4, DHFR, EPX, TPO, MPO, DPYD, NQO1, AKR1C1, CYP19A1 | 6.90E-10 |
|  | GO:0050482~arachidonic acid secretion | 8 | 5.83E-12 | PLA2G10, PLA2G2A, PLA2G1B, PLA2G2C, PLA2G2E, PLA2G2D, PLA2G5, PLA2G2F | 1.03E-09 |
|  | GO:0036149~phosphatidylinositol acyl-chain remodeling | 7 | 3.57E-11 | PLA2G10, PLA2G2A, PLA2G1B, PLA2G2E, PLA2G2D, PLA2G5, PLA2G2F | 5.02E-09 |
|  | GO:0036148~phosphatidylglycerol acyl-chain remodeling | 7 | 8.21E-11 | PLA2G10, PLA2G2A, PLA2G1B, PLA2G2E, PLA2G2D, PLA2G5, PLA2G2F | 9.64E-09 |
|  | GO:0036150~phosphatidylserine acyl-chain remodeling | 7 | 8.21E-11 | PLA2G10, PLA2G2A, PLA2G1B, PLA2G2E, PLA2G2D, PLA2G5, PLA2G2F | 9.64E-09 |
|  | GO:0032355~response to estradiol | 10 | 1.52E-10 | CASP3, CASP9, PTGS2, CASP8, TH, MBD4, CAT, OGG1, NQO1, GSTP1 | 1.53E-08 |
|  | GO:0036152~phosphatidylethanolamine acyl-chain remodeling | 7 | 5.84E-10 | PLA2G10, PLA2G2A, PLA2G1B, PLA2G2E, PLA2G2D, PLA2G5, PLA2G2F | 5.14E-08 |
|  | GO:0006979~response to oxidative stress | 10 | 8.46E-10 | XPA, LPO, PXDN, PTGS2, EPX, PTGS1, TPO, MPO, PRDX5, OGG1 | 6.62E-08 |
|  | GO:0036151~phosphatidylcholine acyl-chain remodeling | 7 | 1.27E-09 | PLA2G10, PLA2G2A, PLA2G1B, PLA2G2E, PLA2G2D, PLA2G5, PLA2G2F | 8.95E-08 |
|  | GO:0006654~phosphatidic acid biosynthetic process | 7 | 6.79E-09 | PLA2G10, PLA2G2A, PLA2G1B, PLA2G2E, PLA2G2D, PLA2G5, PLA2G2F | 4.34E-07 |
|  | GO:0006805~xenobiotic metabolic process | 8 | 3.71E-08 | CYP1B1, SULT1A1, PTGS1, SULT1A3, SULT1A2, NQO1, GSTP1, AKR1C1 | 2.18E-06 |
|  | GO:0016042~lipid catabolic process | 8 | 6.78E-08 | PLA2G10, PLA2G2A, PLA2G1B, PLA2G2C, PLA2G2E, PLA2G2D, PLA2G5, PLA2G2F | 3.67E-06 |
|  | GO:0046135~pyrimidine nucleoside catabolic process | 5 | 9.47E-08 | TYMP, UPP1, UPP2, DPYS, DPYD | 4.76E-06 |
|  | GO:0006644~phospholipid metabolic process | 7 | 1.01E-07 | PLA2G10, PLA2G2A, PLA2G1B, PLA2G2C, PLA2G2E, PLA2G2D, PLA2G5 | 4.76E-06 |
|  | GO:0045471~response to ethanol | 8 | 2.92E-07 | TYMS, SLC6A3, CASP8, TH, CAT, OGG1, NQO1, GSTP1 | 1.29E-05 |
|  | GO:0007568~aging | 9 | 4.61E-07 | TYMS, CYP1A1, CASP9, CASP7, SLC6A3, MPO, CAT, OGG1, NQO1 | 1.91E-05 |
|  | GO:0008202~steroid metabolic process | 6 | 9.95E-07 | AKR1C4, CYP1B1, CYP1A1, SULT1A1, SULT1A3, SULT1A2 | 3.89E-05 |
|  | GO:0045008~depyrimidination | 4 | 4.00E-06 | TDG, MBD4, OGG1, NTHL1 | 1.48E-04 |
|  | GO:0042493~response to drug | 10 | 5.17E-06 | LCN2, TYMS, CASP3, CYP1A1, PTGS2, SLC6A3, PMS2, CAT, COMT, OGG1 | 1.82E-04 |
|  | GO:0032496~response to lipopolysaccharide | 8 | 5.87E-06 | CASP3, CYP1A1, CASP9, PTGS2, CASP8, TH, MPO, COMT | 1.97E-04 |
|  | GO:0006584~catecholamine metabolic process | 4 | 5.97E-06 | SULT1A1, SULT1A3, SULT1A2, COMT | 1.91E-04 |
|  | GO:0046677~response to antibiotic | 5 | 9.65E-06 | CASP3, CYP1A1, CASP9, CASP8, TP53 | 2.95E-04 |
|  | GO:0051923~sulfation | 4 | 1.17E-05 | SULT1A1, SULT1A3, SULT1A2, SULT1C2 | 3.42E-04 |
|  | GO:0006284~base-excision repair | 5 | 1.39E-05 | XPA, TP53, TDG, OGG1, NTHL1 | 3.92E-04 |
|  | GO:0050427~3'-phosphoadenosine 5'-phosphosulfate metabolic process | 4 | 3.18E-05 | SULT1A1, SULT1A3, SULT1A2, SULT1C2 | 8.60E-04 |
|  | GO:0032025~response to cobalt ion | 3 | 2.62E-04 | CASP3, CASP9, CASP8 | 6.80E-03 |
|  | GO:0009635~response to herbicide | 3 | 3.65E-04 | LCN2, CYP1A1, TH | 9.14E-03 |
|  | GO:0009636~response to toxic substance | 5 | 4.59E-04 | XPA, TYMS, CYP1B1, NQO1, GSTP1 | 1.11E-02 |
|  | GO:0009308~amine metabolic process | 3 | 4.86E-04 | CYP1A1, SULT1A1, SULT1C2 | 1.13E-02 |
|  | GO:0009812~flavonoid metabolic process | 3 | 4.86E-04 | CYP1A1, SULT1A1, SULT1A3 | 1.13E-02 |
|  | GO:0008635~activation of cysteine-type endopeptidase activity involved in apoptotic process by cytochrome c | 3 | 6.23E-04 | CASP3, CASP9, CASP7 | 1.40E-02 |
|  | GO:0043066~negative regulation of apoptotic process | 9 | 6.52E-04 | GCG, CASP3, TP53, MPO, PRDX5, CAT, OGG1, NQO1, GSTP1 | 1.42E-02 |
|  | GO:0042416~dopamine biosynthetic process | 3 | 7.76E-04 | DDC, SLC6A3, TH | 1.64E-02 |
|  | GO:0043525~positive regulation of neuron apoptotic process | 4 | 7.92E-04 | CASP3, CASP9, TP53, NQO1 | 1.63E-02 |
|  | GO:0008210~estrogen metabolic process | 3 | 9.46E-04 | CYP1B1, SULT1A1, COMT | 1.89E-02 |
|  | GO:0043097~pyrimidine nucleoside salvage | 3 | 1.13E-03 | TYMP, UPP1, UPP2 | 2.19E-02 |
|  | GO:0042542~response to hydrogen peroxide | 4 | 1.30E-03 | CASP3, HBA2, CAT, HBB | 2.45E-02 |
|  | GO:0071407~cellular response to organic cyclic compound | 4 | 1.99E-03 | CASP3, CYP1B1, CYP1A1, CASP8 | 3.62E-02 |
|  | GO:0097194~execution phase of apoptosis | 3 | 2.04E-03 | CASP3, CASP7, CASP8 | 3.61E-02 |
|  | GO:0080111~DNA demethylation | 3 | 2.04E-03 | TET3, TDG, TET1 | 3.61E-02 |
|  | GO:0033189~response to vitamin A | 3 | 2.58E-03 | TYMS, CYP1A1, CAT | 4.45E-02 |
| **CC** | GO:0005829~cytosol | 34 | 4.21E-08 | TH, UPP1, PRDX5, UPP2, DPYS, COMT, TYMS, TYMP, TYR, CASP3, AKR1C4, CASP9, SULT1A1, CASP7, CASP8, EDC3, SULT1A3, SULT1A2, SULT1C2, CAT, NQO1, HBB, AKR1C1, DDC, HMBS, TP53, HBA2, LCN2, DHFR, TOP3A, DPYD, GSTP1, TOP3B, PLA2G2F | 4.25E-06 |
|  | GO:0005739~mitochondrion | 19 | 2.34E-06 | CYP1B1, CYP1A1, TH, TP53, PRDX5, COMT, SDHA, TYMS, CRY2, CASP9, CASP8, TPO, YJEFN3, MPO, CAT, OGG1, CRY1, NTHL1, GSTP1 | 1.18E-04 |
| **MF** | GO:0004601~peroxidase activity | 9 | 2.23E-14 | PXDN, PTGS2, EPX, PTGS1, TPO, MPO, PRDX5, HBA2, HBB | 5.24E-12 |
|  | GO:0020037~heme binding | 13 | 5.08E-13 | LPO, PXDN, CYP1B1, PTGS2, CYP1A1, PTGS1, HBA2, EPX, MPO, TPO, CAT, HBB, CYP19A1 | 5.97E-11 |
|  | GO:0004623~phospholipase A2 activity | 8 | 4.61E-11 | PLA2G10, PLA2G2A, PLA2G1B, PLA2G2C, PLA2G2E, PLA2G2D, PLA2G5, PLA2G2F | 3.61E-09 |
|  | GO:0003684~damaged DNA binding | 7 | 2.73E-07 | XPA, REV1, CRY2, POLH, TP53, TDG, OGG1 | 1.60E-05 |
|  | GO:0005506~iron ion binding | 9 | 2.78E-07 | LCN2, TET3, CYP1B1, CYP1A1, TH, HBA2, HBB, TET1, CYP19A1 | 1.30E-05 |
|  | GO:0019825~oxygen binding | 6 | 1.64E-06 | CYP1B1, CYP1A1, TH, HBA2, HBB, CYP19A1 | 6.41E-05 |
|  | GO:0004062~aryl sulfotransferase activity | 4 | 1.59E-05 | SULT1A1, SULT1A3, SULT1A2, SULT1C2 | 5.34E-04 |
|  | GO:0097153~cysteine-type endopeptidase activity involved in apoptotic process | 4 | 2.06E-05 | CASP3, CASP9, CASP7, CASP8 | 6.06E-04 |
|  | GO:0008146~sulfotransferase activity | 4 | 5.21E-04 | SULT1A1, SULT1A3, SULT1A2, SULT1C2 | 1.35E-02 |
|  | GO:0047498~calcium-dependent phospholipase A2 activity | 3 | 6.33E-04 | PLA2G2A, PLA2G1B, PLA2G5 | 1.48E-02 |
|  | GO:0019104~DNA N-glycosylase activity | 3 | 7.90E-04 | TDG, MBD4, NTHL1 | 1.67E-02 |
|  | GO:0030983~mismatched DNA binding | 3 | 9.62E-04 | TDG, PMS2, MLH1 | 1.87E-02 |
|  | GO:0016712~oxidoreductase activity, acting on paired donors, with incorporation or reduction of molecular oxygen, reduced flavin or flavoprotein as one donor, and incorporation of one atom of oxygen | 3 | 1.82E-03 | CYP1B1, CYP1A1, CYP19A1 | 3.23E-02 |
|  | GO:0004497~monooxygenase activity | 4 | 1.94E-03 | TYR, CYP1B1, CYP1A1, CYP19A1 | 3.21E-02 |
|  | GO:0004197~cysteine-type endopeptidase activity | 4 | 2.24E-03 | CASP3, CASP9, CASP7, CASP8 | 3.46E-02 |

| **Supplementary Table S4: The enriched KEGG pathways which are significantly associated with target proteins.** | | | | |
| --- | --- | --- | --- | --- |
| **KEGG Pathway** | **Count** | **P Value** | **Genes** | **Benjamini corrected P value** |
| **hsa00592:alpha-Linolenic acid metabolism** | 8 | 1.62E-09 | PLA2G10, PLA2G2A, PLA2G1B, PLA2G2C, PLA2G2E, PLA2G2D, PLA2G5, PLA2G2F | 1.96E-07 |
| **hsa00590:Arachidonic acid metabolism** | 10 | 3E-09 | PTGS2, PLA2G10, PTGS1, PLA2G2A, PLA2G1B, PLA2G2C, PLA2G2E, PLA2G2D, PLA2G5, PLA2G2F | 1.81E-07 |
| **hsa00591:Linoleic acid metabolism** | 8 | 5.12E-09 | PLA2G10, PLA2G2A, PLA2G1B, PLA2G2C, PLA2G2E, PLA2G2D, PLA2G5, PLA2G2F | 2.06E-07 |
| **hsa04975:Fat digestion and absorption** | 8 | 4.69E-08 | PLA2G10, PLA2G2A, PLA2G1B, PLA2G2C, PLA2G2E, PLA2G2D, PLA2G5, PLA2G2F | 1.42E-06 |
| **hsa00565:Ether lipid metabolism** | 8 | 1.33E-07 | PLA2G10, PLA2G2A, PLA2G1B, PLA2G2C, PLA2G2E, PLA2G2D, PLA2G5, PLA2G2F | 3.21E-06 |
| **hsa01100:Metabolic pathways** | 28 | 2.71E-06 | PTGS2, TH, PTGS1, UPP1, UPP2, DPYS, COMT, TYMS, TYR, TYMP, AKR1C4, MCEE, PLA2G1B, TPO, CYP19A1, DDC, PLA2G10, CYP1A1, HMBS, SDHA, DHFR, PLA2G2A, PLA2G2C, DPYD, PLA2G2E, PLA2G2D, PLA2G5, PLA2G2F | 5.47E-05 |
| **hsa04972:Pancreatic secretion** | 8 | 1.96E-05 | PLA2G10, PLA2G2A, PLA2G1B, PLA2G2C, PLA2G2E, PLA2G2D, PLA2G5, PLA2G2F | 3.39E-04 |
| **hsa00564:Glycerophospholipid metabolism** | 8 | 2.26E-05 | PLA2G10, PLA2G2A, PLA2G1B, PLA2G2C, PLA2G2E, PLA2G2D, PLA2G5, PLA2G2F | 3.42E-04 |
| **hsa05204:Chemical carcinogenesis** | 7 | 8.21E-05 | CYP1B1, CYP1A1, PTGS2, SULT1A1, SULT1A3, SULT1A2, GSTP1 | 1.10E-03 |
| **hsa04270:Vascular smooth muscle contraction** | 8 | 8.66E-05 | PLA2G10, PLA2G2A, PLA2G1B, PLA2G2C, PLA2G2E, PLA2G2D, PLA2G5, PLA2G2F | 1.05E-03 |
| **hsa03460:Fanconi anemia pathway** | 6 | 0.000112 | REV1, POLH, TOP3A, PMS2, MLH1, TOP3B | 1.23E-03 |
| **hsa00140:Steroid hormone biosynthesis** | 6 | 0.000173 | AKR1C4, CYP1B1, CYP1A1, COMT, AKR1C1, CYP19A1 | 1.74E-03 |
| **hsa00350:Tyrosine metabolism** | 5 | 0.00027 | DDC, TYR, TH, TPO, COMT | 2.51E-03 |
| **hsa00980:Metabolism of xenobiotics by cytochrome P450** | 6 | 0.000544 | AKR1C4, CYP1B1, CYP1A1, GSTP1, AKR1C1, DHDH | 4.69E-03 |
| **hsa00983:Drug metabolism - other enzymes** | 5 | 0.000782 | TYMP, UPP1, UPP2, DPYS, DPYD | 6.29E-03 |
| **hsa00240:Pyrimidine metabolism** | 6 | 0.00221 | TYMS, TYMP, UPP1, UPP2, DPYS, DPYD | 1.66E-02 |
| **hsa04210:Apoptosis** | 5 | 0.0024 | CASP3, CASP9, CASP7, CASP8, TP53 | 1.69E-02 |
| **hsa03410:Base excision repair** | 4 | 0.00328 | TDG, MBD4, OGG1, NTHL1 | 2.19E-02 |
| **hsa04014:Ras signaling pathway** | 8 | 0.00436 | PLA2G10, PLA2G2A, PLA2G1B, PLA2G2C, PLA2G2E, PLA2G2D, PLA2G5, PLA2G2F | 2.74E-02 |
| **hsa00380:Tryptophan metabolism** | 4 | 0.00568 | DDC, CYP1B1, CYP1A1, CAT | 3.39E-02 |
